# Acaricidal activity of *Foeniculum vulgare* against *Rhipicephalus annulatus* is mainly dependent on its constituent from trans-anethone

**DOI:** 10.1371/journal.pone.0260172

**Published:** 2021-12-02

**Authors:** Shawky M. Aboelhadid, Waleed M. Arafa, Abdel-Azeem S. Abdel-Baki, Atalay Sokmen, Saleh Al-Quraishy, Ahmed O. Hassan, Asmaa A. Kamel

**Affiliations:** 1 Parasitology Department, Faculty of Veterinary Medicine, Beni-Suef University, Beni-Suef, Egypt; 2 Zoology Department, Faculty of Science, Beni-Suef University, Beni-Suef, Egypt; 3 Department of Plant Production and Technologies, Faculty of Agriculture and Natural Sciences, Konya Food and Agriculture University, Konya, Turkey; 4 Zoology Department, College of Science, King Saud University, Riyadh, Saudi Arabia; 5 Department of Medicine, Washington University School of Medicine, St. Louis, Missouri, United States of America; Tocklai Tea Research Institute, INDIA

## Abstract

Globally, the economic losses due to hard ticks infestation and the control of the associated diseases have been calculated at USD $13.9–18.7 billion per year. The economic impact is related to its direct damage to the skins, blood loss, anemia, severe immunological reactions and indirect losses that related to the effects of hemoparasites, cost of treatment for clinical cases and expenses incurred in the control of ticks. The current study evaluated the acaricidal activities of fennel *Foeniculum vulgare* essential oil and its main components; trans-anethole and fenchone; against *R*. *annulatus*. GC–MS analysis revealed that this oil contained 16 components representing 99.9% of the total identified compounds with E-anethole being the predominant component(64.29%), followed by fenchone (9.94%). The fennel oil and trans-anethole showed significant acaricidal activities. The LC_50_ of the fennel oil was attained at concentrations of 12.96% for adult ticks and 1.75% for tick larvae meanwhile the LC_50_ of trans-anethole was reached at concentrations of 2.36% for adult tick and 0.56% for tick larvae. On the contrary, fenchone showed no any significant adulticidal activities and its LC_50_ attained at a concentration of 9.11% for tick larvae. Regarding repellence activities, trans-anethole achieved 100% repellency at the concentration of 10% while fennel showed 86% repellency at the same concentration. Fenchone showed no repellency effect. Treatment of larvae with fennel, trans-anethole, and fenchone LC_50_ concentrations significantly inhibited the acetylcholinesterase activity. Meanwhile, glutathione s-transferase activity was significantly decreased in fennel treated larvae but no significant effect was found in the larvae of trans-anethole and fenchone groups. These results indicate that the acaricide effect of fennel oil may attributed to its high content of trans-anethole. This was supported by potent adulticidal, larvicidal, and repellency effects of trans-anethole against *Rhipeciphalus annulatus* tick and therefore it could be included in the list of acaricide of plant origin.

## Introduction

Cattle tick is a major threat to cattle production and health condition globally. Ticks infestation causes significant loss in meat, milk, and leather production [1]. Tick-borne diseases to cattle as babesiosis and anaplasmosis are also the major health obstacles to effective livestock production [2]. Chemical acaricides are the most common approach for tick control. This strategy of control has several issues on public health hazards through residues and acaricides resistance [3]. Now, plant metabolites and essential oils are considered a suitable alternatives for ticks’ control [4, 5] as it is safe for public health and the environment [6].

Fennel; *Foeniculum vulgare* Mill., is a biennial and aromatic plant widespread in the Mediterranean region and Central Europe [7]. Fennel has been traditionally used for multi-medical purposes as antispasmodic, anti-inflammatory, diuretic, analgesic, and antioxidant remedies [8, 9]. The essential oil of the fennel contains trans-anethole and fenchone as major constituents with some other minor compounds such as methyl chavicol, p-cymene, eugenol, limonene, α-pinene, 1,8-cineole, γ-terpinene, linalool, mycerene, camphor, and α-terpinol [10]. The acaricidal activity of *F*. *vulgare* essential oil was proved against *Varroa destructor*, a major pest of honey bees, *Apis mellifera* L. [11], females of *T*. *urticae* [12], the spotted spider mite, *Tetranychus urticae* [13], and dust dwelling mites, *Dermatophagoides farinae* and *Dermatophagoides pteronyssinus* [14]. Also, it was found to have insecticidal activity against *Melophagus ovinus* sheep ked [15], and larvicidal activity against *Culex pipiens* mosquito [16]. Moreover, Lucca et al. [17] reported 70% mortality rate in aphid nymphs treated with 1% fennel oil.

The present study was therefore suggested to investigate the role of trans-anethole/fenchone in the acaricide activity of fennel essential oil against *Rhipeciphalus annulatus*.

## Materials and methods

### Fennel seeds and isolation of oil

Seeds of *Foeniculum vulgare* were purchased from local market of Konya, Turkey. The seeds were dried overnight in oven at 80°C. Then, the dried seeds were ground with mortar and pestle and the resulted powder was stored in dark at 4°C. One hundred gram of seed powder were hydrodistilled in a Clevenger’s type apparatus for 6 h, to obtain yellow colored oil (yield 3.4%), with specific odor and sharp taste. This crude oil was dried over anhydrous sodium sulphate to remove the traces of moisture then stored in sterilized dark vial in a refrigerator at 4°C until use [18].

### GC-MS analysis of *Foeniculum vulgare* essential oil

The chemical analysis of fennel essential oil (EO) was carried following the method of Adams [18]. The analysis of the EO was performed using a Trace Ultra Gas Chromatographer coupled with a DSQ II Mass Spectrometer (Thermo Scientific). The chromatographic separation of the constituents was accomplished on a TR-5MS (30 m x 0.25 mm x 0.25 μm) capillary column (Thermo Scientific) with a temperature program from 60°C to 250°C by a rate of 3°C min^-1^, and flow rate of helium fixed at 1 mL min^-1^. Injector and MS transfer line temperatures were set at 220°C and 250°C, respectively. Samples were prepared by the dilution of 1 mg of EO in 1 mL of acetone, 1 μL of the diluted sample was injected manually, using splitless mode. The MS was operating in EI mode at 70 eV, the ion source temperature was 240°C, whereas mass spectra were acquired in the scan mode for mass range 35–400. The identification of the compounds was based on the comparison of their relative retention indexes and mass spectra with corresponding data recoreded in literature’s and instrument’s databases (Adams Book 07, Nist 98, Xcalibur). A series of n-alkanes (C8 –C24) was used for the determination of the Relative Retention Index. Relative % percentages of the compounds were obtained electronically from area percent data.

### Chemicals

**Fig 1:**
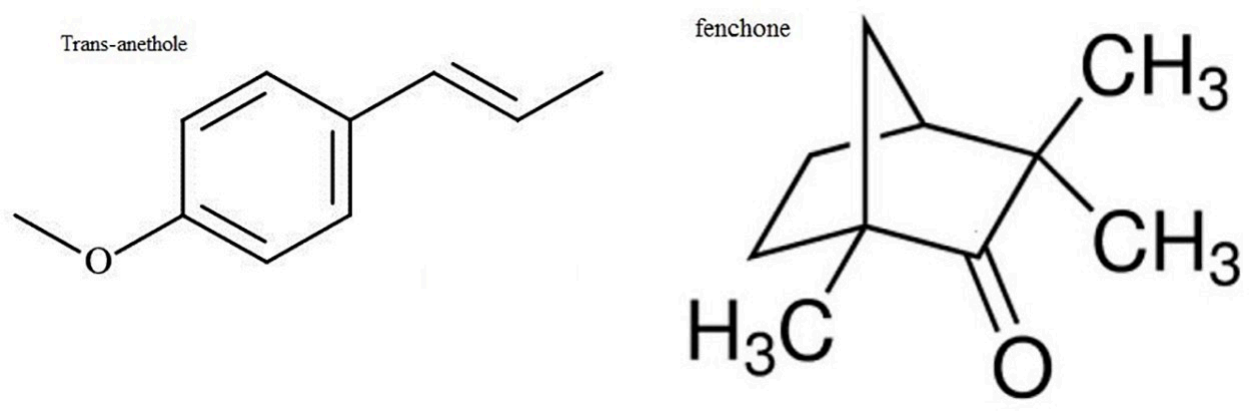
Structure of main components trans-anethole and fenchone.

Trans-anethole and fenchone were purchased from Sigma Alderich. These compound were of analytical standard grade (Fig 1).

### Preparation of the tested concentrations

Five concentrations (10, 5, 2.5, 1.25 and 0.625% volume by volume) were prepared from the fennel essential oil, trans-anethole and fenchone by dissolving in ethyl alcohol 70%.

### Ticks, eggs and larvae

**Fig 2:**
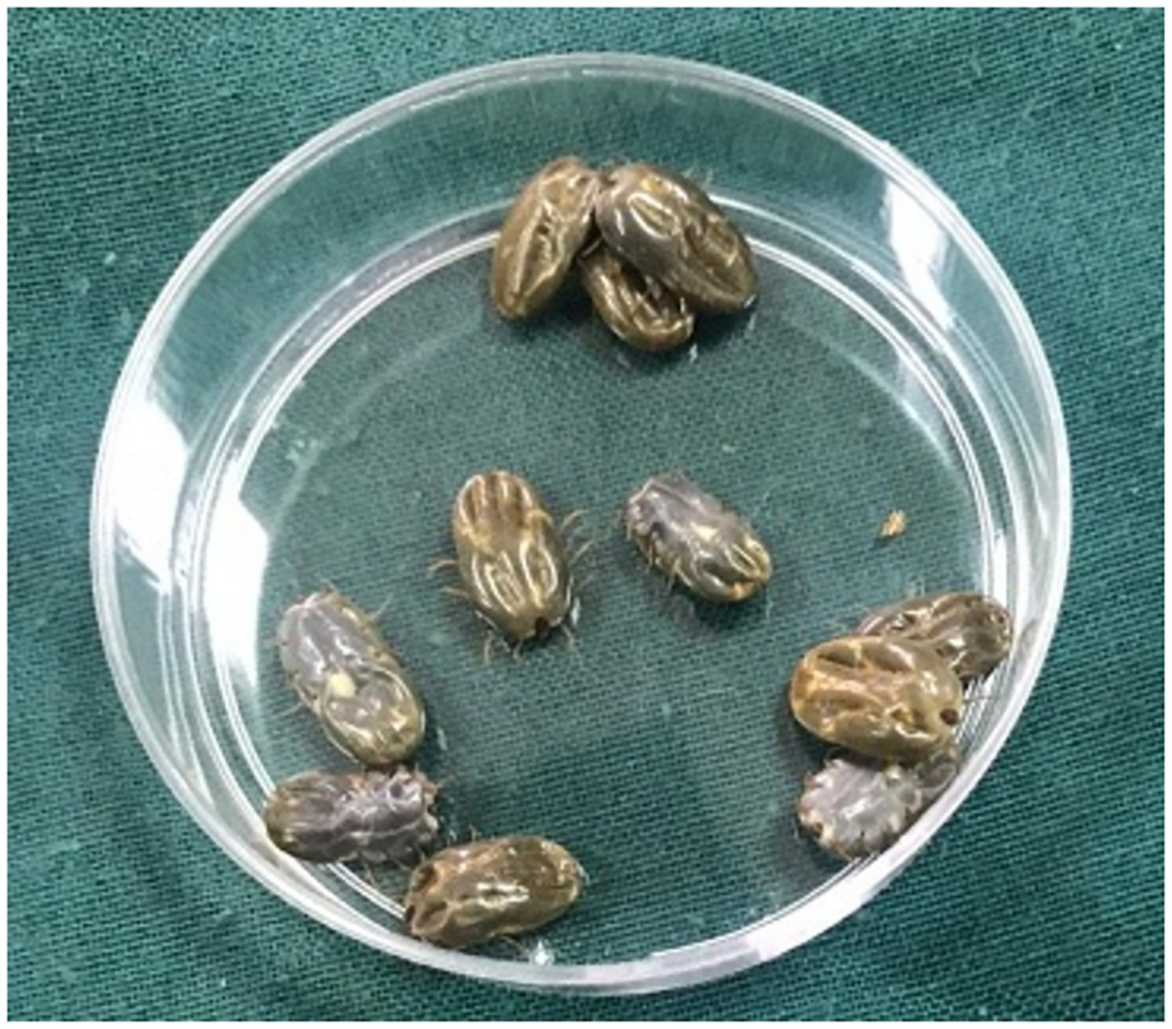
Adult engorged female *Rhipicephalus annulatus* (before application of any treatment).

Adult fully engorged females of *R*. *annulatus* (Fig 2) were collected from naturally infested cattle in different villages in Beni-Suef province, middle Egypt and south to Cairo (Coordinates: 29°04′N 31°05′E)) with at least 3 weeks of previous exposure to any acaricide. The collected ticks were transported to the Parasitology Lab, Faculty of Veterinary Medicine, Beni-Suef University. The ticks were identified according to Estrada-Peña et al. [19]. Part from these ticks was used for adult immersion test while the other part was incubated under laboratory conditions at 27 ± 1.5 °C and 70–80% relative humidity (RH) [20] to obtain eggs, and then larvae that used in the further bioassays.

### Adult Immersion Test (AIT)

The adult immersion test was carried out according to Drummond et al. [20] and FAO [21]. Five replicates were done for each concentration. Ticks in the control group were treated with ethanol 70%. Each replicate contained a group of ten cleaned, healthy, engorged female ticks, with homogeneous weight and size were immersed in 10 ml of one of each dilution of the oil solution in a 7cm diameter petri dish with occasional gentle agitation, at room temperature (approximately 25 8°C). After 2 min the solution was discarded, female ticks were removed and gently dried on paper toweling. The treated ticks were kept in BOD incubator at a temperature of 27±2°C and a relative humidity of 80±10% for oviposition. The deposited eggs of the treated ticks were collected at day 7 post application (PA) and weighed. The mortality rate was also estimated at day 7 PA. Mortality % = [Number of dead tick in a treated group—Number of dead tick in the control group] / Total number of treated ticks × 100.

### Larvicidal activity

Larval packet technique (LPT) with a modification of Matos et al. [22] was applied. By brush, about one hundred larvae of ten days’ age were placed on the center of 7×7 cm filter papers then 100 μL of the tested solutions was added then closed to form packets. The control group was treated with ethanol (70%). Five replicates were performed for each concentration. Packets were examined after 24 h to record mortality rates. Larvae with no motion were considered dead.

### Repellent activity

This bioassay relies upon the vertical migration behavior of tick’s larvae as elucidated by Wanzala et al. [23] with slight modification. The device consisted of two aluminum rods (0.7 × 15 cm), filter paper (7 × 7cm) treated with 200 μL (covering approximately area 28 cm^2^) of the different treatments. The treated filter paper was clipped to one rod, on the other rode, a filter paper was treated with ethanol 70% acted as a negative control. Another, third rode contained a positive control filter paper treated with standard repellent, DEET (N,N-diethyl- 3-methylbenzamide), at concentration of 7.5% [24]. Nearly 30 larvae of *R*. *annulatus* of ten days’ age were placed at the base of each rod then were observed the rods after 15 min, and after one hour. The repellence activity was followed up for 4 hours post application. Ticks larvae that were found on upper of the treated filter paper were considered not repelled while those at the base of the treated filter paper, naked part of the glass rod were considered repelled. This test was performed five times for each concentration. $\text{The}\;\text{repellence}\;(\%)=\frac{\text{The}\;\text{number}\;\text{of}\;\text{larvae}\;\text{on}\;\text{the}\;\text{negative}\;\text{control}-\text{The}\;\text{number}\;\text{of}\;\text{larvae}\;\text{on}\;\text{the}\;\text{treated}}{\text{The}\;\text{number}\;\text{of}\;\text{larvae}\;\text{on}\;\text{the}\;\text{negative}\;\text{control}}\times 100$.

### Anti-acetylcholinesterase (AChE) activity

The acetylecholinestrase was extracted according to Cardoso et al. [25]. Briefly, larvae treated with the LC_50_ of the tested materials. To extract the AChE, the treated larvae were macerated using a mortar and pestle for 5 min in sodium phosphate buffer (100 mM, pH 7.0, containing Triton X- 100, and protease inhibitor mix (1:5 larva weight: buffer volume). This extract was left for 30 min at 4°C, and then was centrifuged at 4°C for 30 min at 10000 rpm. The supernatant was collected and stored at 4°C. The activity of the extracted AChE was estimated according to Ellman et al. [26] with modification of Li et al. [27]. The inhibition percentage of AChE enzyme was calculated by comparison with the negative control as follows: AChE inhibition (%) = 100 − [(As / Ac) × 100], where: As = AChE activity for treated larvae; Ac = Negative control. The larvae in the control group was treated with deltamethrin (1mL/L) while the larvae in negative control group was treated with ethyl alcohol 70%.

### Oxidative and antioxidant biomarkers

Lipid peroxidation (malondialdehyde) (MDA) in the homogenate of the treated larvae was assessed colorimetrically according to the method of Preuss et al. [28]. The colour produced after the reaction of MDA with thiobarbituric acid was measured spectrophotometrically at 532 nm. The estimation of Glutathione (GSH) levels in homogenate of the treated larvae was following the method of Beutler et al. [29]. DTNB with glutathione (GSH) will form a yellow-coloured compound which is directly proportional to the amount of GSH and can be measured at 405 nm.

### Statistics

Statistical analysis of data was performed using Statistical Package for Social Science (SPSS for Windows (IBM), version 22, Chicago, USA). ANOVA tests and subsequent Duncan’s multiple range tests were applied to determine the differences between means. Data were presented as means and the values considered significant at *P < 0*.*05*. The effective concentration (LC_50_) with 95% Confidence Interval (LC _95_%) was calculated (SPSS version 22).

## Results

### Yield and chemical composition of the essential oil

Hydrodistillation of the fennel seeds provided a pale yellow-colored essential oil with a 3.4% (v/w) yield. The oil’s odor was typical of anethol. GC–MS and GC-FID analyses also confirmed this observation. The resulted oil contained 16 components representing 99.9% of the total identified compounds (Table 1), among which E-anethole (64.29%) and fenchone (9.94%) were the predominant ones.

**Table 1:** GC-Mass of fennel essential oil.

KI experimental	Compound	%Area
935	*α*-Pinene	4.33
980	*β*-Pinene	0.11
989	Myrcene	0.26
1006	*α*-Phellandrene	1.58
1026	p-Cymene	0.18
1031	Limonene	7.31
1061	*γ*-Terpinene	0.27
1090	Fenchone	6.94
1154	Camphor	0.01
1198	Methyl chavicol	3.85
1246	Carvone	0.03
1255	(Z)-Anethole	0.06
1257	p-Anisaldehyde	0.31
1285	(E)-Anethole	74.43
1382	Anisyl methyl ketone	0.12
1434	α-trans-Bergamotene	0.08
	Total	99.87
traces: < 0,09%		

### Adulticidal activity

**Fig 3:**
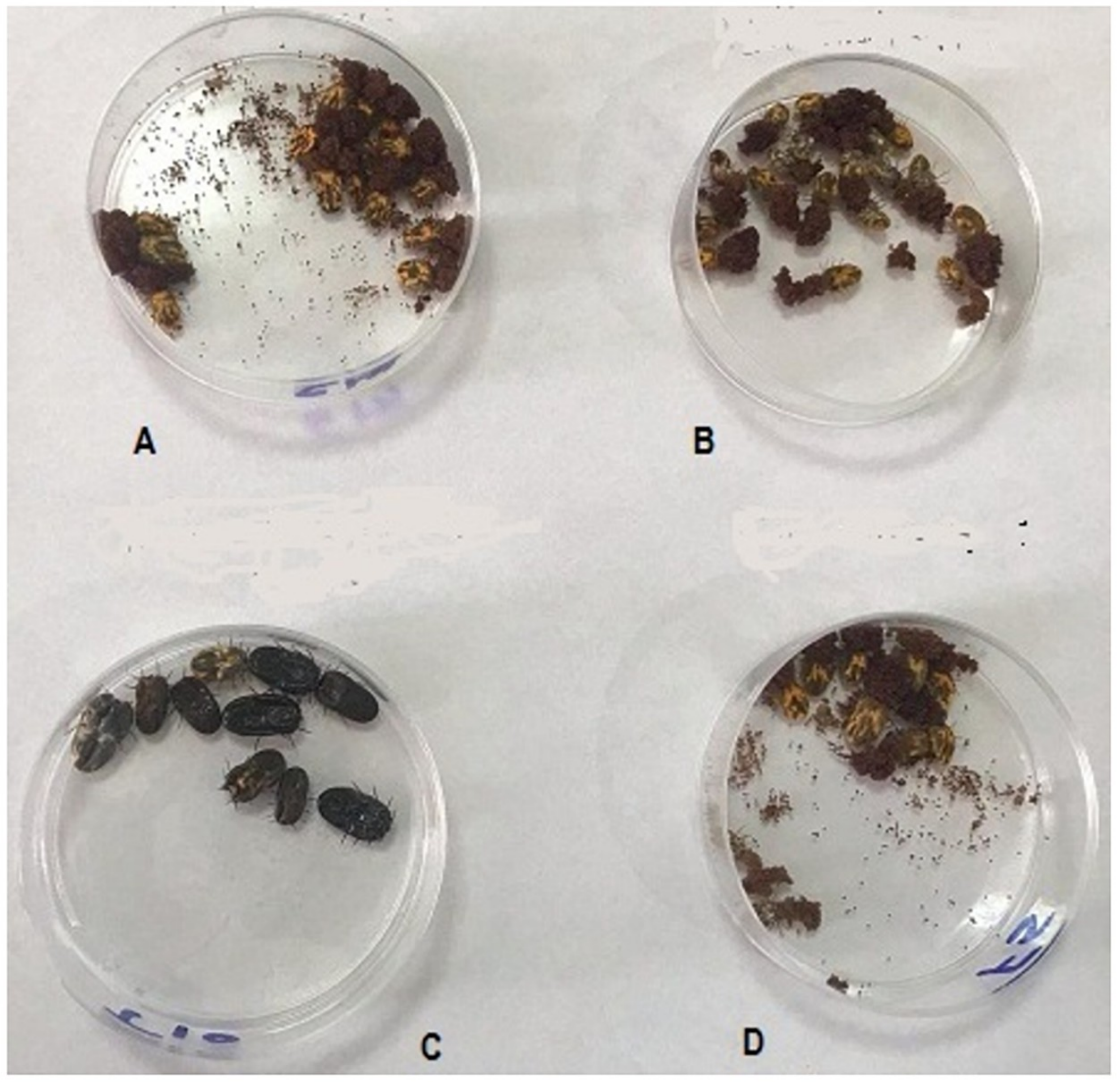
Adult *Rhipicephalus annulatus* ticks treated by different compounds at day 7 post treatments; A. Treated ticks by 70% ethyl alcohol deposited eggs, B. treated ticks by deltamethrin (1mL/L) deposited eggs, C. Dead ticks treated by trans-anethole 10%, D. Treated ticks by fenchone 10% deposited eggs.

*Foeniculum vulgare* (fennel) essential oil and its main constituents showed variable degrees of acaricidal activity against *R*. *annulatus* ticks. Fennel showed significant adulticidal activity only at concentration of 10% with 30% mortality and LC_50_ attained at a concentration of 12.96%. Also, fennel 10% inhibited the percent of egg production to 66.67%. Trans-anethole meanwhile showed significant acaricidal activity especially at concentrations of 5 and 10% with tick mortality rate reached to 82.67 and 100% respectively and LC_50_ achieved at a concentration of 2.36% (Fig 3). Also, trans-anethole 5% reduced the percent of egg production to 91.02%. On the contrary, fenchone didn’t show any significant adulticidal activities at concentrations of ≤ 10% (Table 2).

**Table 2:** Adulticidal and lethal concentrations (LC_50_, LC_90_) of Fennel oil, trans-anethole and fenchone against *R*. *annulatus* adult ticks.

Treatment	Conc. %	Mortality % M±SE	% inhibition of egg production	LC_50_ (95% CL)	LC_90_ (95% CL)	χ2 (df = 3)	*P*
*Fennel oil*	0.625	0.00±0.00^b^	5.77	12.96 (11.21–17.62)	23.49 (17.39–18.24)	2.13	0.546
	1.25	0.00±0.00^b^	17.94				
	2.5	0.00±0.00^b^	20.52				
	5	0.00±0.00^b^	35.9				
	10	30.00±5.77^a^	66.67				
Trans-anethole	0.625	0.00±0.00^e^	39.75	2.36 (2.13–2.60)	5.49 (4.77–6.56)	7.74	0.052
	1.25	22.33±1.45^d^	58.98				
	2.5	54.00±2.08^c^	66.04				
	5	82.67±1.45^b^	91.02				
	10	100.00±0.00^a^	100				
Fenchone	0.625	0.00±0.00	4.48	NA	NA	NA	NA
	1.25	0.00±0.00	5.77				
	2.5	0.00±0.00	8.98				
	5	0.00±0.00	11.54				
	10	2.33±0.67	19.23				
Deltamethrin	uL/L	10.00±5.77	38.46				
Ethyl alcohol	70%	0.00±0.00	0				

Means within the same column followed by different superscripts are significantly different (Duncan’s multiple range test: *P* ≤ 0.05). *X*^*2*^ chi square. (df) degree of freedom. NA = not available.

### Larvicidal activity

Trans-anethole revealed significant larvicidal activity with 55% larval mortality rate at the concentration of 0.625% and 100% larval mortality rate was reached at the concentration of 2.5% with LC_50_ attained at a concentration of 0.56% (Table 3). Meanwhile, fenchone showed significant larvicidal activity only at the concentration of 10% with 58.33% mortality rate and LC_50_ reached at the concentration of 9.11% (Table 3).

**Table 3:** Larvicidal activity, and LC_50_, and LC_90_ of Fennel oil, trans-anethole and fenchone against larvae of *R*. *annulatus*.

Treatment	Mortality rate (Mean ±SE) Concentration					LC50 (95% CL)	LC90 (95% CL)	χ2 (df = 3)	*p*
	0.625%	1.25%	2.5%	5%	10%				
Fennel oil	6.67±1.67^c^	16.67±3.33^b^	75.00±2.89^b^	100±0.00^a^	100±0.00^a^	1.75 (1.21–2.59)	3.407 (2.368–8.981)	14.728	0.002
Trans-anethole	55.00 ±2.89^a^	85.00 ± 2.89^a^	100.00 ± 0.00^a^	100.00 ± 0.00^a^	100.00 ± 0.00^a^	0.56 (0.43–0.67)	1.65 (1.402–2.06)	0.631	0.631
Fenchone	0.00 ± 0.00^d^	16.67 ± 1.67^b^	19.33 ± 0.67^c^	23.00 ± 1.00^b^	58.33 ± 4.41^b^	9.11 (4.16–34621.45)	59.334 (14.078–1.796E+12	1.726	0.000
Deltamethrin uL/mL	18.67±0.88^b^	18.67±0.88^b^	18.67±0.88^c^	18.67±0.88^b^	18.67±0.88^b^	NA	NA	NA	NA
Ethyl alchol (70%)	11.67±1.67^c^	11.67±1.67^b^	11.67±1.67^d^	11.67±1.67^c^	11.67±1.67^c^	NA	NA	NA	NA

Means within the same column followed by different superscripts are significantly different (Duncan’s multiple range test: *P* ≤ 0.05).

NA = not available.

### Repellent activity

**Fig 4:**
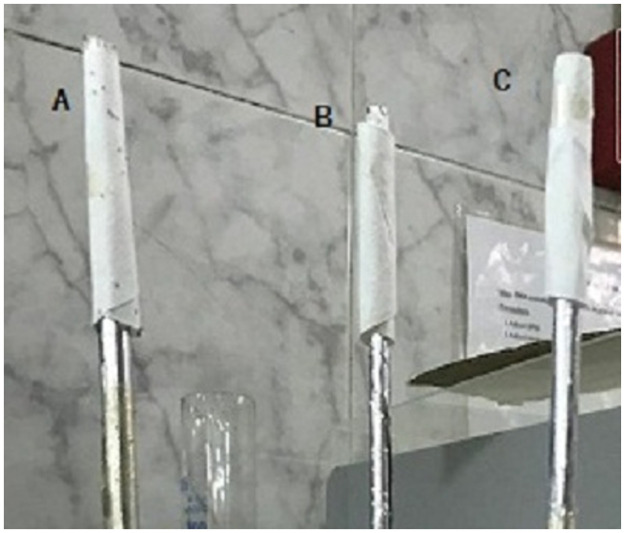
Repellency technique (Road vertical method) at the first hour post application: A. Control negative ethyl alcohol 70% showed tick larvae at the top of the filter paper, B. Trans-anethole treated filter paper with no larvae at the top. C. Control positive treated filter paper by DEET showed no larvae at the top.

**Fig 5:**
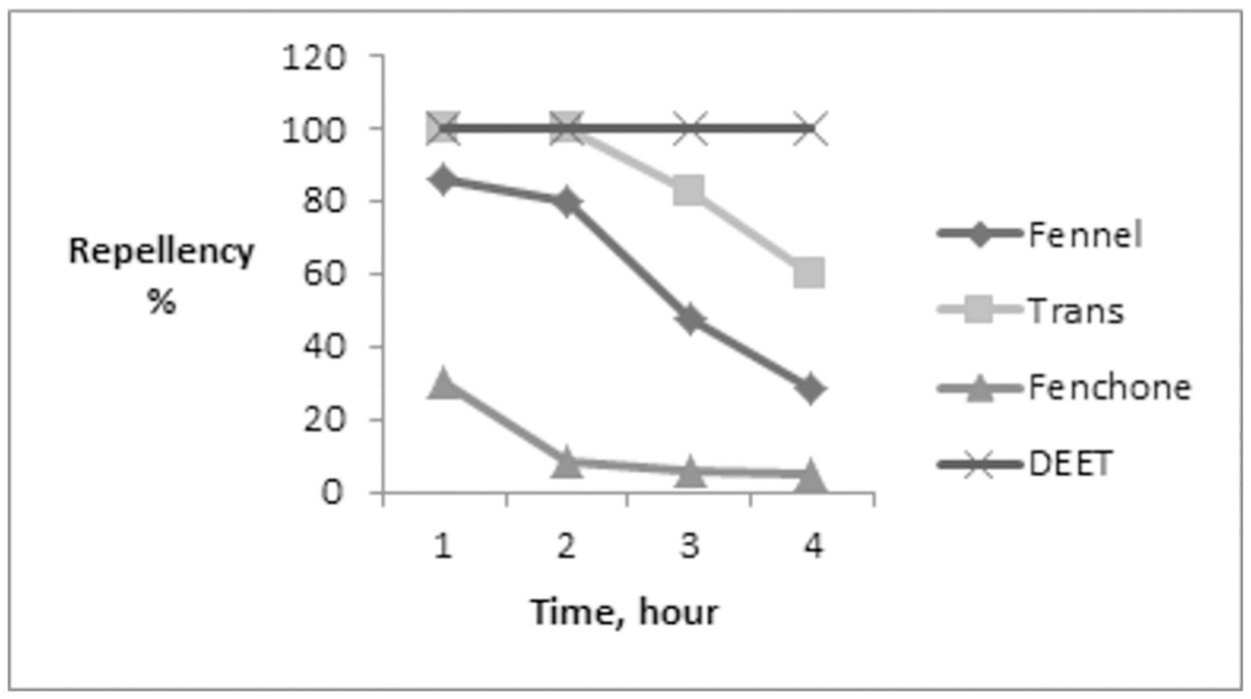
Repellency efficacy (%) of fennel essential oil, trans-anethole and fenchone against the *R*. *annulatus* larvae at concentration of 10% with application of DEET 7.5% as control positive.

Fennel oil showed the highest repellency of 86% at a concentration of 10% at the first hour and repellency declined to 29% at the 4^th^ hour (Fig 4). While trans-anethole achieved 100% repellency similar to DEET in positive control group at a concentration of 10% at the first two hours then repellency decreased to 59.67% at the 4^th^ hour which significantly lower than that of DEET. Fenchone showed a weak repellency even at the concentration of 10% (Table 4, Fig 5). The repellency of DEET was 100% even after 4 hours.

**Table 4:** Repellency percentages of Fennel oil, trans-anethole and fenchone against *R*. *annulatus* larvae.

Treatment	Concentration %	Repellency % (Mean ±SE)			
		1^st^ hour	2^nd^ hour	3^rd^ hour	4^th^ hour
*Foeniculum vulgare*	0.625	25.00±2.89^d^	9.67±1.20^e^	0.00±0.00	0.00±0.00
	1.25	31.00±2.08^cd^	23±1.15^d^	9.67±0.88^d^	6.00±1.15^d^
	2.5	33.00±2.00^c^	28±1.86^c^	20±1.45^c^	14.00±1.67^c^
	5	64.00±2.00^b^	41±2.08^b^	34±2.08^b^	20.00±1.15^b^
	10	86.00±2.31^a^	80±1.15^a^	47.67±1.45^a^	29.00±2.08^a^
Trans-anethole	0.625	30.33±1.45^a^	15.00±1.73^b^	5.67±1.201^c^	0.00±0.00^d^
	1.25	48.33±4.49^a^	31.67±1.86^b^	25.00±1.73^c^	16.33±1.45^d^
	2.5	67.33±1.86^a^	38.00±4.04^b^	24.67±2.03^c^	12.33±0.88^d^
	5	85.33±2.403^a^	83.00±2.89^a^	62.67±1.76^b^	32.00±2.08^c^
	10	100.00±0.00^a^	100.00±0.00^a^	83.00±1.15^b^	59.67±5.93^c^
Fenchone	0.625	0.00±0.00^c^	0.00±0.00^b^	0.00±0.00^b^	0.00±0.00
	1.25	0.00±0.00^c^	0.00±0.00^b^	0.00±0.00^b^	0.00±0.00
	2.5	0.00±0.00^c^	0.00±0.00^b^	0.00±0.00^b^	0.00±0.00
	5	14.67±2.03^a^	7.67±0.88^a^	6.00±1.15^a^	0.00±0.00
	10	30.33±3.93^b^	8.67±1.33^a^	6.33±1.45^a^	0.00±0.00
DEET (control positive)	7.5%	100.00±0.00	100.00±0.00	100.00±0.00	100.00±0.00
Ethyl alcohol (control negative)	70%	0.00±0.00	0.00±0.00	0.00±0.00	0.00±0.00

Means within the same column followed by different superscripts are significantly different (Duncan’s multiple range test: *P* ≤ 0.05).

### Acetylcholinesterase inhibition and oxidative enzymes of treated *R*. *annulatus* tick larvae

Treatment of *R*. *annulatus* tick larvae with concentrations of the L_C50_ of fennel oil and its main components; trans-anethole and fenchone significantly inhibited the activity of AchE when compared with that of the control untreated larvae (Table 5). Fennel exhibited the highest inhibition followed by trans-anethole and then fenchone whereas the lowest AchE inhibition was found in the deltamethrin-treated group. Glutathione S-transferases (GST) activity was significantly decreased in fennel treated larvae but no significant difference was found between the deltamethrin treated larvae and trans-anethole treated ones (Table 5). The treatment with fennel EO induced a significant increase in the level of Malondialdehyde (MDA) when compared with the untreated larvae. On contrary, the treatment with trans-anethole and fenchone showed no significant effect on the level MDAwith also, no significant difference was found between the both groups (Table 5).

**Table 5:** Oxidative enzymes and acetylcholinesterase inhibition of treated *R*. *annulatus* tick larvae by fennel and its main components; trans-anethone and fenchone.

Groups	AchE Inhibition (%)	GST (ng)	MDA (μmol/L)
Control untreated	0.00±0.00^e^	2.92±0.01^a^	2.96±0.01^d^
Deltamethrin (mL/L)	15.23±0.14 ^d^	2.43±0.01^c^	3.57±0.01^b^
Fennel	30.00±0.29 ^a^	1.88±0.01^d^	4.23±0.01^a^
Trans-anethole	25.40±0.09 ^b^	2.68± 0.01^c^	3.11±0.05^cd^
Fenchone	18.17±0.08 ^c^	2.43±0.06^b^	3.22±0.13^c^

AchE = acetylcholinesterase, GST = glutathione- S-Transferase, MDA = malondialdehyde.

Means within the same column followed by different superscripts are significantly different (Duncan’s multiple range test: *P* ≤ 0.05).

## Discussion

Synthetic organic acaricides have been widely used to control ticks. The increasing resistance of ticks to these acaricides in addition to the problems associated with their meat and milk residues and potential toxicity on non-target organisms have become significant challenges to the effectiveness of such chemotherapy, however [30, 31]. Accordingly, great efforts are now being devoted to investigate the natural products for acaricide activities, which probable to be a critical attributes and eco-friendly alternatives for ticks control [5, 32–34]. For many years, the acaricidal properties of the plant essential oils have been widely studied against ticks [35]. One of the most attractive features of essential oils is that they are low-risk products [36]. Also, essential oils are eco-friendly [37] and unlike some synthetic insecticides; no bio-magnification has been reported to date [35].

In the present study, fennel EO was extracted from the seeds using standard distillation method as recommended by Bakkali et al. [38] and Piras et al. [39]. GC–MS and GC-FID analyses of the extracted EO revealed that trans-anethole and fenchone were the major constituents similar to those reported by He and Huang [10]. Therefore, herein the adulticidal, the larvicidal, and the repellent activities of fennel EO, trans-anethole and fenchone against *R*. *annulatus* tick were investigated. Fennel EO didn’t show adulticidal activity at concentrations of ≤ 5%, whereas at a concentration of 10% tick mortality was reached to 30% with LC_50_ attained at a concentration of 12.96%. Meanwhile, the adulticidal activities of trans-anethole reached to 100% at a concentration of 10% with LC_50_ accomplished at a concentration of 2.22%. On the contrary, fenchone didn’t show adulticidal activity at any of the tested concentrations. In the same context, Pavela et al. [3] reported a significant adulticidal activity for fennel EO against adult *Musca domestica*. Also, Amizadeh et al. [12] found a similar adulticidal effect for the fennel against adult females of *Tetranychus urticae*. Additionally, several studies reported significant toxicity for the fennel EO to different types of mites e.g. *Tetranychus urticae*, *Dermatophagoides farinae and Dermatophagoides pteronyssinus* [13, 14]. Also, fennel found to have insecticidal activity against the sheep ked *Melophagus ovinus* [15] and the aphid nymphs [17]. Fennel EO was also proven to be highly toxic to two important insect pests of the stored product (*Sitophilus oryzae and Callosobruchus Chinensis*) [40]. According to the available literature so far, there are no reports on the effect of trans-anethole on the adult tick of *R*. *annulatus*.

In the current investigation, all the tested concentrations of fennel EO showed various degrees of larvicidal activity with LC_50_ attained at a concentration of 1.75%. Trans-anethole showed the best larvicidal effect as evidenced by the lowest concentration (0.56%) required to attain the LC50 meanwhile, fenchone showed weakest larvicidal effect as its LC50 reached at a concentration of 9.11%. Our results come in accordance with de Oliveira Souza Senra et al. [41] as they found that the treatment with the highest concentrations of fennel oil (20.0 μl/ml) caused 100% mortality in the larvae of *R*. *microplus* and *D*. *nitens*. Similarly, Chantawee and Soonwera [42] found 100% mortality in the larvae of *Aedes aegypti* treated with *F*. *vulgare* EO at a concentration of 10% with LT_50_ achieved at a concentration of 5%. Also, a similar larvicidal activity was reported for *F*. *vulgare* against the 4^th^ instar larvae of *Ae*. *aegypti*, *Anopheles dirus*, *Anopheles stephensi*, *Culex pipiens* mosquitoes [4, 16, 43, 44].

Regarding the repellency, *F*. *vulgare* EO showed concentration-dependent repellent activity with highest repellency (86%) achieved at the concentration of 10% which was comparably lower than that of the positive control DEET. Trans-anethole achieved 100% repellency at a concentration of 10% at the first two hours while fenchone showed no any repellency effect. Similarly, *F*. *vulgare* showed a significant repellent activity against *Ae*. *aegypti* females [45, 46]. Also, Cosimi et al. [47] found a moderate level of repellency for the fennel EO against *Sitophilus zeamais*. In addition, repellency activity was also observed for anethole-rich fennel EO against the granary weevil *S*. *granarius* L. [48], the fleas [49], and the ants of the genus *Solenopsis* [50]. This study represents the first of its type on repellency activities of fennel and trans-anethole against *R*. *annulatus*.

Plant EO directly affects insect survival and disrupts their physiological processes by provoking tissue damages [51] which might be due to the induction of free radicals after EO administration [52, 53]. Acetylcholinesterase (AchE) is one of the foremost vital hydrolytic enzymes in the insect nervous system that equilibrate neural signal transduction by rapid hydrolyzing of acetylcholine signal in the synaptic cleft [54].

In the present study, all treatments induced inhibition in the AChE activity when compared with that of the control untreated larvae. Similarly, Shahriari et al. [55] noticed an inhibition in the activity of AChE of *E*. *kuehniella* larvae that fed on an artificial diet containing α-pinene, trans-anethole, and thymol. Also, Kim et al. [56] found reduction in the activity of AchE of *S*. *oryzae* after the treatment with α-pinene and trans-anethole. Plant EOs revealed different inhibitory properties for AchE which might be due to differences in their lipophilicity and volatility as well as the inhibition of P450 monooxygenases [57].

Regarding the glutathione, GST activity was significantly decreased in fennel-treated larvae. Meanwhile, trans-anethole showed no significant effect on glutathione activity when compared with that of the control group. These results were in accordance with Shahriari et al. [55] as they demonstrated higher GST activity in the larvae of *E*. *kuehniella* fed on the artificial diet containing α-pinene, trans-anethole, and thymol. Similar results were also obtained when pupae of *D*. *melanogaster* were treated with Azadirachtin [58]. Our data revealed that the Malondialdehyde (MDA) production significantly increased in the larvae treated with fennel EO when compared with that of the untreated larvae. Meanwhile the treatment with trans-anethole showed no significant effect on the level of MDA. These findings were similar to those of Shahriari et al. [55] as they reported increase in the level of MDA in larvae of *E*. *kuehniella* after treatment with mixture of α-pinene, trans-anethole, and thymol while the level of MDA in larvae treated with trans-anethole alone remained with no significant change. Rahimi et al. [59] demonstrated that *P*. *persicaria* Agglutinin induced elevation in the level of MDA in the larvae of *Helicoverpa armigera*. They attributed this elevation to the cytotoxicity induced in the midgut epithelial cells of insects after the treatment with plant-derivative compounds [59].

In conclusions, trans-anethole represented 64.29% of total fennel oil components and showed adulticidal, larvicidal and repellent activity better than its precursor fennel oil and /or other main component; fenchone. Consequently, the acaricide activity of fennel oil is may linked mainly to the presence of trans-anethole.

Nonetheless, 10% trans-anethole is the effective concentration which is still high and its application in field is limited. Also, the natural product is not persist in the environment and easily degraded by photo-oxidation temperature and the solvents used [60]. Therefore, further experiments are needed to affirm its suitability to the field application and modification is also needed in its formulations to be more stable in the practical application.

## Supporting information

S1 Video A video showing adult immersion technique. (MP4)

S2 Video A video showing repellency technique. (MP4)
